# Correlated impurity complex in the asymmetric tunneling contact: an ideal system to observe negative tunneling conductivity

**DOI:** 10.1038/s41598-019-52095-8

**Published:** 2019-11-04

**Authors:** N. S. Maslova, P. I. Arseyev, V. N. Mantsevich

**Affiliations:** 1 Quantum Technology Center and Quantum electronics department, Faculty of Physics, Lomonosov Moscow State University, 119991 Moscow, Russia; 2 P.N. Lebedev Physical Institute RAS, 119991 Moscow, Russia; 3 Quantum Technology Center and Semiconductors and Cryoelectronics department, Faculty of Physics, Lomonosov Moscow State University, 119991 Moscow, Russia

**Keywords:** Physics, Condensed-matter physics, Semiconductors

## Abstract

We studied theoretically electron transport through the impurity complex localized between the tunneling contact leads by means of the generalized Keldysh diagram technique. The formation of multiple well pronounced regions with negative tunneling conductivity in the I-V characteristics was revealed. The appearance of negative tunneling conductivity is caused by the presence of both strong Coulomb correlations and the asymmetry of tunneling rates, which lead to the blockade of the electron transport through the system for a certain values of applied bias. The developed theory and obtained results may be useful for the application of impurity (dopant) atoms as a basic elements in modern nanoelectronic circuits.

## Introduction

Nowadays electron transport through impurity complexes attracts strong attention. It occurs because impurity (dopant) atoms are considered to be both promising candidates for the implementation in semiconductor nanoelectronic devices^1–4^ and model systems for quantum transport phenomena investigation^5–8^. Application of individual atoms as building blocks of nanoelectronic devices is very perspective, as they have a stable well defined electronic structure. Individual atoms embedded in the semiconductor medium allow to fabricate unique single-atom single-electron tunneling devices as prototypes of quantum logic gates^9^, quantum bits^10–12^, charge pumps and turnstiles^13–16^, etc.

One of the most vital properties for electronic components functioning is the presence of negative tunneling conductivity^17^, which was observed for the first time in a highly doped tunneling diods^18^. Later negative tunneling conductivity has been revealed in a quite few low temperature experiments of electron transport through molecules^1,2,19–23^, dopant atoms^24^ and quantum dots^25^. For molecular systems the presence of negative tunneling conductivity was demonstrated both at low and room temperatures^1,2,19,21^. Usually, negative tunneling conductivity occurs at the back of sharp current peaks and is explained by the charge transfer between two energy levels which are in resonance at a certain value of applied bias and are out of resonance for other values of applied bias.

One of the first theoretical explanations of negative tunneling conductivity was proposed by Likharev with co-authors^8^. They attributed formation of negative tunneling conductivity with the enhancement of one of the two tunneling barriers of the transistor by the source-drain electric field. This mechanism made it possible to explain experimental results obtained in molecular systems^19,20^. Another possible mechanism for the appearance of negative tunneling conductivity in molecular electronic devices was proposed in^22^ and originated from local orbital symmetry matching between an electrode and a molecule in a single molecular electronic device. However, mechanisms considered in^8,22^ could not explain formation of negative tunneling conductivity in a small size strongly correlated systems^25^, where the presence of strong Coulomb interaction plays an important role and should be taken into account. Later it was demonstrated that in a low dimensional strongly correlated structures negative tunneling conductivity arises due to the presence of non-equilibrium Coulomb interaction^26–28^. Inter-particle correlations should be carefully taken into account as they could drastically affect the local charge distribution in the vicinity of impurity complexes in nanometer-size tunneling junctions. Moreover, interacting impurities is one of the most promising systems for the analysis of Coulomb correlations influence on the properties of the electron transport^28–34^.

Electronic circuits based on the individual impurities are recently under active investigation, therefore understanding the role of strong inter-particle correlations in such systems and analysis of their influence on the electron transport properties would be an important milestone on the way of single atom nanoelectronic devices creation. Here we theoretically analyze electron transport through the impurity cluster with strong Coulomb correlations between localized electrons by means of the generalized Keldysh diagram technique. We reveal that the presence of strong Coulomb correlations in the asymmetric tunneling contact (coupling strength between the impurity complex and metallic contacts strongly differ) leads to the formation of multiple well pronounced regions with negative tunneling conductivity in the I-V curves. This finding is very promising in the sense of dopant atoms application as basic elements in modern nanoelectronic circuits.

## Model System and Theoretical Approach

### Model system

Further we will consider a model two-level impurity system localized between metallic tunneling contact leads. Two-level system is usually applied for the analysis of electron transport through the correlated impurities. Single electron energy levels *ε*_*i*_ could be associated with two different impurities or they could both correspond to the single impurity. We would like to mention that energy spectrum of correlated impurity complex could be rather complicated depending on the strength of coupling between the impurities and the substrate, the value of inter-particle interaction, type of the substrate, etc. However, the situation when only two levels of the impurity complex contribute significantly to the electron transport could be realized by means of the applied bias and gate voltage tuning. Two-level system is relevant, when only two energy levels from the whole impurity complex spectrum are localized in the energy gap *E*_*F*_ < *ε*_*i*_ < *E*_*F*_−*eV*. Inequality means that only these two levels contribute to the tunneling current. The Hamiltonian of the two-level system could be written as

1 $$\hat{H}={\hat{H}}_{imp}+{\hat{H}}_{lead}+{\hat{H}}_{tun}.$$

Hamiltonian ${\hat{H}}_{imp}$ describes impurity complex and includes on-site and inter-site Coulomb interaction in the Hubbard form:

2 $${\hat{H}}_{imp}=\sum _{i\sigma }\,{\varepsilon }_{i}{\hat{n}}_{i}^{\sigma }+\sum _{ij\sigma \sigma \text{'}}\,{U}_{ij}^{\sigma \sigma \text{'}}{\hat{n}}_{i}^{\sigma }{\hat{n}}_{j}^{\sigma \text{'}}.$$

Further ${\hat{n}}_{i\sigma }={\hat{c}}_{i\sigma }^{+}{\hat{c}}_{i\sigma }$ is localized electron occupation numbers operator and operator ${\hat{c}}_{i\sigma }$ destroys electron with spin *σ* at the energy level *ε*_*i*_. ${U}_{ij}^{\sigma \sigma \text{'}}$ is the Coulomb repulsion between localized electrons. Further we assume that on-site Coulomb repulsion ${U}_{ii}^{\sigma \sigma \text{'}}$ exceeds inter-site Coulomb repulsion ${U}_{ij}^{\sigma \sigma \text{'}}$. Such an assumption corresponds to the situation, when impurity state localization radius is smaller than the distance between the impurities. Part ${\hat{H}}_{lead}$ describes continuous spectrum states in the metallic tunneling contact leads

3 $${\hat{H}}_{lead}=\sum _{k\sigma }\,{\varepsilon }_{k}{\hat{c}}_{k\sigma }^{+}{\hat{c}}_{k\sigma }+\sum _{p\sigma }\,({\varepsilon }_{p}-eV){\hat{c}}_{p\sigma }^{+}{\hat{c}}_{p\sigma },$$

where indices *k* and *p* label continuous spectrum states in the different leads of the tunneling junction. Operators ${\hat{c}}_{k(p)}^{+}/{\hat{c}}_{k(p)}$ correspond to the creation/annihilation of the electrons in the continuous spectrum states *k*(*p*) and *eV* is the applied bias voltage. Tunneling part ${\hat{H}}_{tun}$ is responsible for coupling between the imputrity complex and the leads

4 $${\hat{H}}_{tun}=\sum _{ki\sigma }\,{t}_{ki}{\hat{c}}_{k\sigma }^{+}{\hat{c}}_{i\sigma }+\sum _{pi\sigma }\,{t}_{pi}{\hat{c}}_{p\sigma }^{+}{\hat{c}}_{i\sigma }+h.c.$$

Tunneling transfer amplitudes *t*_*k*(*p*)*i*_ between continuous spectrum states in the leads and the two-level system are considered to be independent on momentum and spin. Further we will analyze only elastic tunneling processes. As it was shown in^35^ inelastic contribution to the tunneling current in the frame of adiabatic scheme at low temperature is much smaller, than the elastic one. Thus, it is sufficient to consider only elastic tunneling and take into account Coulomb correlations to obtain negative tunneling conductivity in asymmetric tunneling contact for impurity atoms with deep levels. It was demonstrated that inelastic processes lead to the additional peculiarities in the I-V characteristics, tunneling conductivity and current noise spectrum but it is not the scope of this paper^36^. In this work we analyze the effect of the two-level system intrinsic properties on the electron transport, while effects caused by the leads, such as the band width effect or the image charge effect, are not discussed.

**Figure 1:**
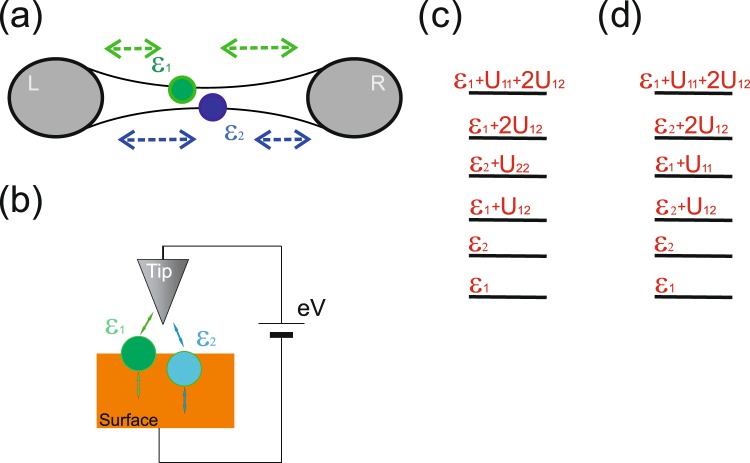
(**a**) Scheme for the I-V curves measurements in the nanobridge geometry. (**b**) Scheme for the I-V curves measurements in the STM/STS experiments. (**c**) Scheme of the two-level system energy levels contributing to the tunneling current for different values of applied bias (Γ_*L*2_ >> Γ_*L*1_ ≃ Γ_*R*1_ >> Γ_*R*2_). (**d**) Scheme of the two-level system energy levels contributing to the tunneling current for different values of applied bias (Γ_*L*1_ >> Γ_*L*2_ ≃ Γ_*R*2_ >> Γ_*R*1_).

We would like to mention, that among the most promising systems for the appearance of negative tunneling conductivity are impurity complexes or QDs embedded in a semiconductor matrix. The peculiarities of electron transport through such systems could be analyzed by means of the tunneling measurements. The most evident technique is the scanning tunneling microscopy/spectroscopy (STM/STS)^37,38^ (see Fig. 1b for the scheme of the measurements geometry). The ability to image individual dopant atoms combined with scanning tunneling spectroscopy allows to directly study the transport mechanisms through the impurities. It is important, that impurity atoms could be localized directly on the semiconductor surface or several nanometers below the surface. In STM measurements semiconductor substrate and a tip of the scanning tunneling microscope form leads of the tunneling contact. In modern tunneling experiments typical current values can be of the order of 10 pA–10 nA. Tunneling rates could vary from 10 *μ*eV to 100 meV depending both on the dopant atoms position and on the distance between the STM tip and the semiconductor surface. Another promising possibility to study properties of the electron transport through the impurity complexes deals with tunneling through a nanobridge where the effective island is formed by the dopant cluster^4,39^ – single electron transistor (see Fig. 1a for the scheme of the measurements geometry). The device is typically a gated silicon nanobridge with a thickness and width of 20 nm. Fabrication of such structures makes it possible to control the number of impurities and their spatial positions during the sample preparation procedure. For the single electron transistor tunneling current is typically about 0.1–10 nA and tunneling rates could be varied in a wide range by means of the gate voltage tuning. Among the most interesting materials applicable for realization of controllable single electron transport is graphene. Electron transport peculiarities in graphene could be connected not only with the presence of localized states but also with particular energy spectrum and non-trivial density of states^40^. In comparison with conventional tunneling devices the tunneling carriers in graphene cross only a few atomic layers, offering the prospect of ultra-fast transit times and small size of the device.

### Green’s functions formalism

Several theoretical approaches are usually used for analysis of the electron transport through the atomic-scale devices. Among them are approaches based on the Hubbard operators^41,42^, pseudo-particle approach^43,44^, well known non-equilibrium Keldysh diagram technique or equations of motion formalism. The problem of Hubbard operators approach deals with the non-trivial commutation rules for Hubbard operators, so it is rather difficult to consider high order correlations only by means of this approach. That is why in^41,42^ authors tried to combine non-equilibrium diagram technique with theoretical approach based on the Hubbard operators. Theoretical scheme based on the pseudo-particles seems to be more convenient in some cases as it allows to generalize Keldysh diagram technique with full account of constraint on the total number of pseudo-particles^43,44^. Here we tried to avoid all these difficulties introducing non-equilibrium Green functions in the operator form and performing averaging over localized electron states at the very last stage in the equations of motion. It allows to obtain the spectrum weight of Green functions with account of high order correlations, which are necessary for the proper treatment of strong Coulomb interaction. Such an approach gives us the possibility to analyze carefully electron transport through the two-level strongly correlated structure and to reveal multiple well pronounced areas with negative differential conductivity in the I-V curves.

Let us introduce operators for retarded ${\hat{G}}_{\mu \nu }^{R\sigma }(t,t^{\prime} )$, advanced ${\hat{G}}_{\mu \nu }^{A\sigma }(t,t^{\prime} )$ and lesser ${\hat{G}}_{\mu \nu }^{ < \sigma }(t,t^{\prime} )$ Green functions as a products of fermion creation and annihilation operators^45^:

5 $$\begin{array}{ccc}{\hat{G}}_{\mu \nu }^{R\sigma }(t,t^{\prime} ) & = & -\,i\Theta (t-t^{\prime} )[{\hat{c}}_{\mu \sigma }(t){\hat{c}}_{\nu \sigma }^{+}(t^{\prime} )+{\hat{c}}_{\mu \sigma }^{+}(t^{\prime} ){\hat{c}}_{\nu \sigma }(t)],\\ {\hat{G}}_{\mu \nu }^{A\sigma }(t,t^{\prime} ) & = & {\hat{G}}_{\mu \nu }^{R\sigma +}(t^{\prime} ,t),\\ {\hat{G}}_{\mu \nu }^{ < \sigma }(t,t^{\prime} ) & = & i{\hat{c}}_{\mu \sigma }^{+}(t){\hat{c}}_{\nu \sigma }({t}^{\text{'}}),\\ {\hat{G}}_{\mu \nu }^{ < \sigma }(t,t) & = & i{\hat{n}}_{\mu \nu }^{\sigma }(t),\end{array}$$

with indexes *ν*, *μ* = *k*, *p*, *i*, *j*. Indexes *i*, *j* describe impurity complex and indexes *k*, *p* correspond to the continuous spectrum states in the leads. Equations of motion for the impurity complex retarded Green functions operators ${\hat{G}}_{ii}^{0R\sigma }$ and ${\hat{G}}_{ii}^{R\sigma }$ have the form:

6 $$\begin{array}{ccc}i\frac{\partial }{\partial t}{\hat{G}}_{ii}^{0R\sigma }-[{\hat{G}}_{ii}^{0R\sigma },{\hat{H}}_{0}] & = & \delta (\tau ),\\ i\frac{\partial }{\partial t}{\hat{G}}_{ii}^{R\sigma }-[{\hat{G}}_{ii}^{R\sigma },\hat{H}] & = & \delta (\tau ),\end{array}$$

where *τ* = *t* − *t*′ and square brackets […] denote the commutator, *δ*(*τ*) is the Dirac delta function and Hamiltonian ${\hat{H}}_{0}$ is given by Eq. (1) without tunneling part. We consider the situation, when non-crossing approximation occurs, so the following ratios between the system parameters are valid Δ*ε*_*i*_/Γ_*i*_ >> 1, (Δ*ε*_*i*_ + *U*_*ij*_)/Γ_*i*_ >> 1. Δ*ε*_*i*_ is the difference between the energies of the single electron levels. In the stationary case total electron occupation numbers are time independent, so one can obtain the Fourier transformed retarded Green function operator ${\hat{G}}_{11}^{R\sigma }(\omega )$ in the following form:

7 $$\begin{array}{ccc}{\hat{G}}_{11}^{R\sigma }(\omega ) & = & \frac{(1-{\hat{n}}_{1}^{-\sigma })(1-{\hat{n}}_{2}^{-\sigma })(1-{\hat{n}}_{1}^{\sigma })}{\omega -{\varepsilon }_{1}-i{\Gamma }_{1}}+\frac{{\hat{n}}_{1}^{-\sigma }(1-{\hat{n}}_{2}^{-\sigma })(1-{\hat{n}}_{1}^{\sigma })}{\omega -{\varepsilon }_{1}-{U}_{1}-i{\Gamma }_{1}}+\sum _{\sigma \text{'}}\,\frac{(1-{\hat{n}}_{1}^{-\sigma }){\hat{n}}_{2}^{\sigma \text{'}}(1-{\hat{n}}_{2}^{\sigma \text{'}})}{\omega -{\varepsilon }_{1}-{U}_{12}-i{\Gamma }_{1}}\\  &  & +\,\sum _{\sigma \text{'}}\,\frac{{\hat{n}}_{1}^{\sigma }{\hat{n}}_{2}^{\sigma \text{'}}(1-{\hat{n}}_{2}^{\sigma \text{'}})}{\omega -{\varepsilon }_{1}-{U}_{1}-{U}_{12}-i{\Gamma }_{1}}+\frac{{\hat{n}}_{2}^{\sigma }{\hat{n}}_{2}^{-\sigma }(1-{\hat{n}}_{1}^{-\sigma })}{\omega -{\varepsilon }_{1}-2{U}_{12}-i{\Gamma }_{1}}+\frac{{\hat{n}}_{2}^{\sigma }{\hat{n}}_{2}^{-\sigma }{\hat{n}}_{1}^{-\sigma }}{\omega -{\varepsilon }_{1}-{U}_{1}-2{U}_{12}-i{\Gamma }_{1}}.\end{array}$$

Relaxation rates Γ_1_ = Γ_*L*1_ + Γ_*R*1_ and ${\Gamma }_{Li(Ri)}=\pi \cdot {t}_{k(p)i}^{2}\cdot {\nu }_{0L(R)}$ describe electron transitions between the impurity complex and the tunneling contact leads. *ν*_0*L*(*R*)_ is the continuous spectrum density of states in the leads. Green function operator ${\hat{G}}_{22}^{R\sigma }(\omega )$ could be obtained from Eq. (7) by the following indexes substitution 1 ↔ 2. Imaginary part of the retarded Green function operator given by Eq. (7) after averaging over localized electrons states directly determines the local density of states in one of the impurities depending on the charge and spin configuration of the whole system considering correlation effects. Poles of each Green function give the energy spectrum of the electrons localized in the vicinity of impurity. In the case of half-filling without taking into account second and high order correlation functions and Kondo effect all the states with the different number of electrons demonstrate the same spectral weight. Tunneling through the strongly correlated structure results in the difference between the spectral weights, as now second and high order correlation functions should be taken into account. For fermions the following relations take place ${({\hat{n}}_{i}^{\sigma })}^{2}={\hat{n}}_{i}^{\sigma }$ and ${\hat{n}}_{i}^{\sigma }(1-{\hat{n}}_{i}^{\sigma })=0$, so all the terms in the perturbation series for the retarded Green function with the different numerators vanish in Eq. (7). Total retarded Green function is obtained as a sum of terms with uncorrelated denominators with the particular energies *ε*_*i*_, *ε*_*i*_ + *U*_*i*_, *ε*_*i*_ + *U*_*ij*_ etc., and each term should be multiplied by a proper combination of the electron occupation number operators. Averaging of the lesser Green function operator allows to obtain the occupation numbers of the impurity complex energy levels. Moreover, non-equilibrium Green functions formalism makes it possible to take into account the non-equilibrium distribution of tunneling particles caused by the tunneling current flowing and the finite value of applied bias. The stationary equation of motion for the operator ${\hat{G}}_{ii}^{ < }(\omega )$ after decoupling localized and conduction electron states and averaging over the electron states in the leads has the form:

8 $$\int d\omega {\hat{G}}_{ii}^{\sigma  < }(\omega )\cdot [{\Sigma }_{i}^{R}(\omega )-{\Sigma }_{i}^{A}(\omega )]-\int d\omega {\Sigma }_{i}^{ < }(\omega )\cdot [{\hat{G}}_{ii}^{A}(\omega )-{\hat{G}}_{ii}^{R}(\omega )]=0,$$

where self-energies

9 $$\begin{array}{ccc}{\Sigma }_{i}^{ < }(\omega ) & = & -2i\cdot [{\Gamma }_{Li}{f}_{L}(\omega )+{\Gamma }_{Ri}{f}_{R}(\omega )],\\ {\Sigma }_{i}^{R}(\omega )-{\Sigma }_{i}^{A}(\omega ) & = & -2i\cdot ({\Gamma }_{Li}+{\Gamma }_{Ri}).\end{array}$$

*f*_*L*(*R*)_(*ω*) is the Fermi distribution function for electrons in the lead *L*(*R*). This equation coincides with the Heisenberg equation of motion for occupation number operators ${\hat{n}}_{i}^{\sigma }$, which includes all order correlation functions for the localized electrons. Taking into account ratios ${({\hat{n}}_{i}^{\sigma })}^{2}={\hat{n}}_{i}^{\sigma }$ and ${\hat{n}}_{i}^{\sigma }(1-{\hat{n}}_{i}^{\sigma })=0$ one could obtain closed system of equations for the all orders correlation functions of localized electron occupation numbers by multiplying Eq. (8) on different combinations of occupation numbers.

### Tunneling through the impurity complex

Our goal is to calculate the tunneling current between the leads in the presence of intermediate impurity complex with strong Coulomb correlations. Let us further consider $\hslash =1$ and *e* = 1 elsewhere. It is natural to define the current as the change in the total number of fermion particles in the reservoir per unit of time. Taking the *k* reservoir for definiteness, we have for the variation of the total particle number:

10 $${\hat{I}}_{T}=\sum _{\sigma }\,{\hat{I}}_{k\sigma }=\sum _{k\sigma }\,{\dot{\hat{n}}}_{k\sigma }=\sum _{ki\sigma }\,{t}_{ki}({\hat{c}}_{k\sigma }^{+}{\hat{c}}_{i\sigma }-{\hat{c}}_{i\sigma }^{+}{\hat{c}}_{k\sigma }).$$

Tunneling current operator can be expressed through the non-equilibrium lesser Keldysh Green function operator^45^:

11 $$i{\hat{c}}_{k\sigma }^{+}{\hat{c}}_{i\sigma }={\hat{G}}_{ki}^{ < }(t,t)=\int \frac{d\omega }{2\pi }{\hat{G}}_{ki}^{ < }(\omega ).$$

Considering Eq. (11) an expression for the tunneling current operator between the contact leads in the frequency representation can be obtained by means of the non-equilibrium diagram technique formalism:

12 $${\hat{I}}_{T}=\sum _{ki\sigma }\,{t}_{ki}\int \frac{d\omega }{2\pi }\cdot [{\hat{G}}_{ki}^{ < }(\omega )-{\hat{G}}_{ik}^{ < \sigma }(\omega )].$$

During the averaging procedure over reservoir states we decouple electron operators in the leads from the localized electron operators. Thus all orders correlation functions for the localized electron operators in the impurity complex can be taken into account. Expression (12) for the reduced tunneling current operator (averaged over conduction electron states) could be re-written through the advanced, retarded and lesser impurity Green functions operators

13 $${\langle {\hat{I}}_{T}^{\sigma }\rangle }_{res}=\sum _{i\sigma }\,{\Gamma }_{Li}\int \frac{d\omega }{2\pi }\{[{\hat{G}}_{ii}^{A\sigma }(\omega )-{\hat{G}}_{ii}^{R\sigma }(\omega )].{f}_{L}(\omega )-{\hat{G}}_{ii}^{ < \sigma }(\omega )\}.$$

Considering Eqs. (8) and (9) and using averaging procedure over localized electron states one could re-write Eq. (13) as

14 $${I}_{T}=\sum _{i}\,\frac{4{\Gamma }_{Li}{\Gamma }_{Ri}}{{\Gamma }_{Li}+{\Gamma }_{Ri}}\int d\omega [{G}_{ii}^{A}(\omega )-{G}_{ii}^{R}(\omega )]\cdot [{f}_{L}(\omega )-{f}_{R}(\omega )],$$

The retarded (advanced) Green function is obtained after averaging over localized electron states ${G}_{ii}^{R(A)}(\omega )=\langle {\hat{G}}_{ii}^{R(A)}(\omega )\rangle$. Finally, averaged tunneling current through the impurity complex could be written as

15 $$\begin{array}{ccc}{I}_{T}^{\sigma } & = & \frac{4{\Gamma }_{L1}{\Gamma }_{R1}}{{\Gamma }_{L1}+{\Gamma }_{R1}}\times \{\langle (1-{n}_{1}^{-\sigma })(1-{n}_{2}^{-\sigma })(1-{n}_{1}^{\sigma })\rangle \cdot [{N}_{L}^{\sigma }({\varepsilon }_{1})-{N}_{R}^{\sigma }({\varepsilon }_{1})]\\  &  & +\,\langle {n}_{1}^{-\sigma }(1-{n}_{2}^{-\sigma })(1-{n}_{1}^{\sigma })\rangle \cdot [{N}_{L}^{\sigma }({\varepsilon }_{1}+{U}_{1})-{N}_{R}^{\sigma }({\varepsilon }_{1}+{U}_{1})]\\  &  & +\,\langle \sum _{\sigma \text{'}}\,(1-{n}_{1}^{-\sigma }){n}_{2}^{\sigma \text{'}}(1-{n}_{2}^{\sigma \text{'}})\rangle \cdot [{N}_{L}^{\sigma }({\varepsilon }_{1}+{U}_{12})-{N}_{R}^{\sigma }({\varepsilon }_{1}+{U}_{12})]\\  &  & +\,\langle {n}_{2}^{\sigma }{n}_{2}^{-\sigma }(1-{n}_{1}^{-\sigma })\rangle \cdot [{N}_{L}^{\sigma }({\varepsilon }_{1}+2{U}_{12})-{N}_{R}^{\sigma }({\varepsilon }_{1}+2{U}_{12})]\\  &  & +\,\langle \sum _{\sigma \text{'}}\,{n}_{1}^{\sigma }{n}_{2}^{\sigma \text{'}}(1-{n}_{2}^{\sigma \text{'}})\rangle \cdot [{N}_{L}^{\sigma }({\varepsilon }_{1}+{U}_{1}+{U}_{12})-{N}_{R}^{\sigma }({\varepsilon }_{1}+{U}_{1}+{U}_{12})]\\  &  & +\,\langle {n}_{2}^{\sigma }{n}_{2}^{-\sigma }{n}_{1}^{-\sigma }\rangle \cdot [{N}_{L}^{\sigma }({\varepsilon }_{1}+{U}_{1}+2{U}_{12})-{N}_{R}^{\sigma }({\varepsilon }_{1}+{U}_{1}+2{U}_{12})]\}+(1\leftrightarrow 2),\end{array}$$

where functions *N*_*L*(*R*)_^*σ*^(*X*) depend on the leads properties and have the form

16 $$\begin{array}{rcl}{N}_{L(R)}^{\sigma }(X) & = & \frac{1}{\pi }\int d\omega {f}_{L(R)}^{\sigma }(\omega )\times \frac{{\Gamma }_{i}}{{(\omega -X)}^{2}+{\Gamma }_{i}^{2}},\\ {\Gamma }_{i} & = & {\Gamma }_{Li}+{\Gamma }_{Ri}.\end{array}$$

${f}_{L(R)}^{\sigma }(\omega )$ is the Fermi distribution function of electrons in the tunneling contact leads. Fermi levels in the *L* and *R* leads are shifted on the value of applied bias *eV*, so *f*_*L*_*(ω*) = *f*_*F*_*(ω*) and *f*_*R*_*(ω*) = *f*_*F*_*(ω*−*eV*). Expression (15) describes tunneling through the all electron states of the considered system (single-, two- three and four-electron states are available in the two-level system). Careful analysis of the tunneling processes through the multi-electron states beyond the mean-field approximation leads to the necessity of the second and high order correlation functions calculation between the electron occupation numbers. In the two-level system tunneling current depends on the localized electron correlation functions up to the third order. In the paramagnetic case when electron occupation numbers with the opposite spins have the same value (${n}_{i}^{\sigma }={n}_{i}^{-\sigma }={n}_{i}$) localized electron correlation functions ${K}_{ii}^{\sigma -\sigma }=\langle {n}_{i}^{\sigma }{n}_{j}^{-\sigma }\rangle ={K}_{ii}$, ${K}_{ij}^{\sigma \sigma \text{'}}=\langle {n}_{i}^{\sigma }{n}_{j}^{\sigma \text{'}}\rangle ={K}_{ij}$ and ${K}_{iij}^{\sigma -\sigma \sigma \text{'}}=\langle {n}_{i}^{\sigma }{n}_{i}^{-\sigma }{n}_{j}^{\sigma \text{'}}\rangle ={K}_{iij}$ do not depend on the spin of the electrons and could be found from the closed system of linear equations:

17 $$A\times (\begin{array}{l}{n}_{1}\\ {n}_{2}\\ {K}_{11}\\ {K}_{12}\\ {K}_{22}\\ {K}_{112}\\ {K}_{221}\end{array})=(\begin{array}{c}{N}_{1}^{T}\\ {N}_{2}^{T}\\ 0\\ 0\\ 0\\ 0\\ 0\end{array}),$$

where matrix *A* depends on the functions *Nσ*_*i*_(*ε*_*i*_), *Nσ*_*i*_(*ε*_*i*_ + *U*_*ij*_), *Nσ*_*i*_(*ε*_*i*_ + *U*_*i*_ + *U*_*ij*_), *Nσ*_*i*_(*ε*_*i*_ + 2*U*_*ij*_) and *Nσ*_*i*_(*ε*_*i*_ + *U*_*i*_ + 2*U*_*ij*_). The explicit form of the correlation functions is given in the Appendix section. Expression (15) has a simple form for tunneling through the single electron level

18 $${I}_{T}^{\sigma }=\frac{4{\Gamma }_{L}{\Gamma }_{R}}{{\Gamma }_{L}+{\Gamma }_{R}}\times \{\frac{[1-{N}^{T\sigma }({\varepsilon }_{1}+U)]\cdot [{N}_{L}^{\sigma }({\varepsilon }_{1})-{N}_{R}^{\sigma }({\varepsilon }_{1})]}{1+{N}^{T\sigma }({\varepsilon }_{1})-{N}^{T\sigma }({\varepsilon }_{1}+U)}+\frac{{N}^{T\sigma }({\varepsilon }_{1})\cdot [{N}_{L}^{\sigma }({\varepsilon }_{1}+U)-{N}_{R}^{\sigma }({\varepsilon }_{1}+U)]}{1+{N}^{T\sigma }({\varepsilon }_{1})-{N}^{T\sigma }({\varepsilon }_{1}+U)}\}.$$

The tunneling occupation numbers ${N}_{i}^{T\sigma }(X)$ are

19 $${N}_{i}^{T\sigma }(X)=\frac{{\Gamma }_{Li}{N}_{L}^{\sigma }(X)+{\Gamma }_{Ri}{N}_{R}^{\sigma }(X)}{{\Gamma }_{i}}.$$

Expression (19) directly demonstrates that tunneling current flowing causes non-equilibrium distribution of the carriers in the intermediate structure. The first term in Eq. (18) describes tunneling through the single-electron states, while the second one corresponds to the tunneling current flowing through the two-electron states. For the applied bias being *ε*_1_ < *e* V< *ε*_1_ + *U* occupation numbers *N*^*Tσ*^(*ε*_1_ + *U*) and ${N}_{L(R)}^{\sigma }({\varepsilon }_{1}+U)$ are close to zero, so tunneling current is given only by the first term of Eq. (18). With the increasing of applied bias (*ε*_1_ + *U* < *eV*) tunneling current is given by the electron transport through both single- and two-electron states. Considering the situation when impurity complex energy levels are well defined (the distance between energy levels strongly exceeds their widths $\frac{\Delta {\varepsilon }_{i}}{{\Gamma }_{L(R)i}} > \, > 1$) one could get an expression for the current through the single -electron and two -electron states taking into account Coulomb correlations of localized electrons in all the orders^26–28^. When applied bias is smaller, than multi-electron energy levels *eV* < *min*(*ε*_*i*_ + *U*_*ij*_; *ε*_*i*_ + *U*_*i*_) only single electron states are available for tunneling. Tunneling current through the single electron states reads:

20 $$\begin{array}{c}{I}_{I}^{\sigma }=\frac{\frac{4{\Gamma }_{L1}{\Gamma }_{R1}}{{\Gamma }_{L1}+{\Gamma }_{R1}}\times [{N}_{L}^{\sigma }({\varepsilon }_{1})-{N}_{R}^{\sigma }({\varepsilon }_{1})]\cdot [1-{N}_{2}^{T\sigma }({\varepsilon }_{2})]}{[1+{N}_{1}^{T\sigma }({\varepsilon }_{1})]\cdot [1+{N}_{2}^{T\sigma }({\varepsilon }_{2})]-4{N}_{1}^{T\sigma }({\varepsilon }_{1})\cdot {N}_{2}^{T\sigma }({\varepsilon }_{2})}+\frac{\frac{4{\Gamma }_{L2}{\Gamma }_{R2}}{{\Gamma }_{L2}+{\Gamma }_{R2}}\times [{N}_{L}^{\sigma }({\varepsilon }_{2})-{N}_{R}^{\sigma }({\varepsilon }_{2})]\cdot [1-{N}_{1}^{T\sigma }({\varepsilon }_{1})]}{[1+{N}_{1}^{T\sigma }({\varepsilon }_{1})]\cdot [1+{N}_{2}^{T\sigma }({\varepsilon }_{2})]-4{N}_{1}^{T\sigma }({\varepsilon }_{1})\cdot {N}_{2}^{T\sigma }({\varepsilon }_{2})}.\end{array}$$

Expression for the tunneling current through the two-electron states (*ε*_1_ + *U*_12_ < *eV* < *min*(*ε*_*i*_ + 2*U*_*ij*_)) can be written as:

21 $$\begin{array}{ccl}{I}_{II}^{\sigma } & \simeq  & \frac{4{\Gamma }_{L1}{\Gamma }_{R1}}{{\Gamma }_{L1}+{\Gamma }_{R1}}\times \{[{N}_{L}^{\sigma }({\varepsilon }_{1}+{U}_{12})-{N}_{R}^{\sigma }({\varepsilon }_{1}+{U}_{12})]\cdot {N}_{2}^{T\sigma }({\varepsilon }_{2})[1-{N}_{1}^{T\sigma }({\varepsilon }_{1})][1-{N}_{2}^{T\sigma }({\varepsilon }_{2}+{U}_{2})]\\  &  & +[{N}_{L}^{\sigma }({\varepsilon }_{1}+{U}_{1})-{N}_{R}^{\sigma }({\varepsilon }_{1}+{U}_{1})]\cdot {N}_{1}^{T\sigma }({\varepsilon }_{1})[1-{N}_{2}^{T\sigma }({\varepsilon }_{2})][1-{N}_{2}^{T\sigma }({\varepsilon }_{2}+{U}_{12})]\}+(1\leftrightarrow 2).\end{array}$$

## Results and Discussion

**Figure 2:**
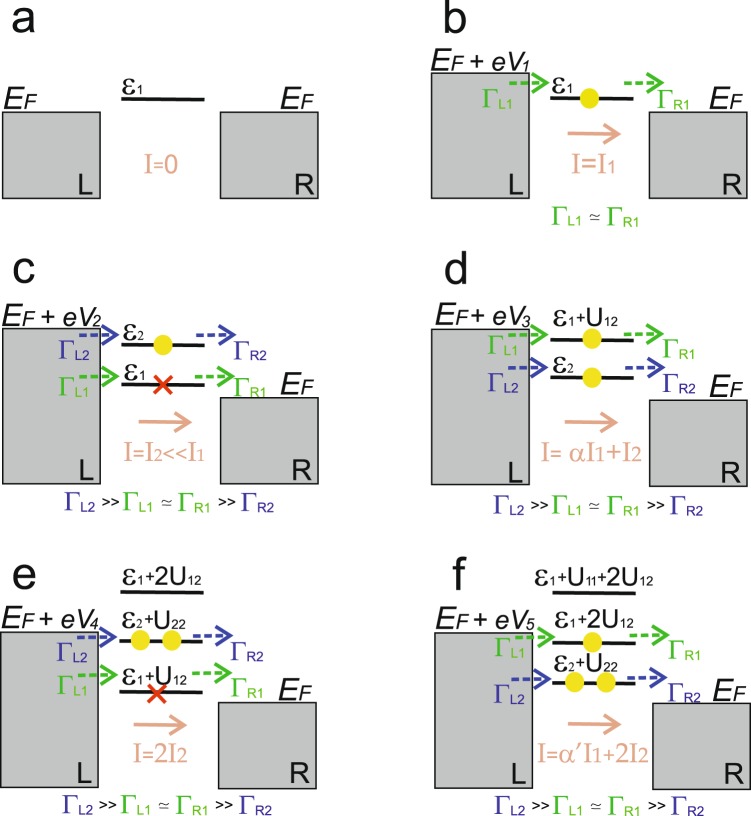
Scheme of the tunneling processes through the two -level system in the asymmetric contact with the following relation between the tunneling rates Γ_*L*2_ >> Γ_*L*1_ ≃ Γ_*R*1_ >> Γ_*R*2_. The value of applied bias *eV*_*i*_ increases from panel (b) to panel (f). Only energy levels contributing to the tunneling current are depicted for each value of applied bias. Full scheme of the two-level system energy levels contributing to the tunneling current for the  different values of applied bias is shown in Fig. 1c.

**Figure 3:**
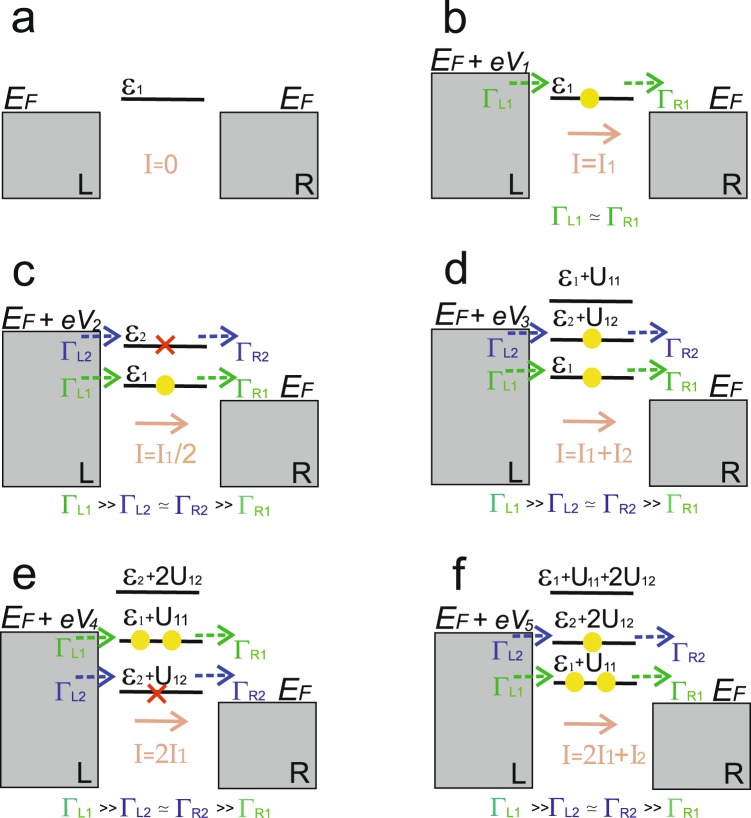
Scheme of the tunneling processes through the two -level system in the asymmetric contact with the following relation between the tunneling rates Γ_*L*1_ >> Γ_*L*2_ ≃ Γ_*R*2_ >> Γ_*R*1_. The value of applied bias *eV*_*i*_ increases from panel (b) to panel (f). Only energy levels contributing to the tunneling current are depicted for each value of applied bias. Full scheme of the two-level system energy levels contributing to the tunneling current for the different values of applied bias is shown in Fig. 1d.

We will further analyze electron transport in the asymmetric tunneling contact. The energy spectrum of the two-level system including electron states contributing to the tunneling current depending on the ratio between the tunneling rates is shown in Fig. 1c,d. Further to avoid difficulties with the results presentation we will demonstrate only energy levels, which fall into the range *E*_*F*_ < *ε* < *E*_*F*_ + *eV* and, consequently, contribute to the tunneling current. We would like to stress, that Figs. 2 and 3 represent an increase in the voltage applied between the electrodes, not moving of the impurity complex energy spectrum. Let us start from the situation, when the single electron energy level *ε*_2_ is asymmetrically coupled to the leads. In this case the following relation between the tunneling rates is realized:

22 $${\Gamma }_{L2} > \, > {\Gamma }_{L1}\simeq {\Gamma }_{R1} > \, > {\Gamma }_{R2}.$$

For the small values of applied bias 0 < *eV* < *ε*_*i*_ (see Fig. 2a) occupation numbers ${N}_{i}^{T}({\varepsilon }_{i})$ are close to zero, consequently, following Eq. (20) tunneling current is negligibly small (*I*_*T*_ → 0). With the increasing of applied bias the single -electron impurity level with the smallest energy starts to be localized between *E*_*F*_ and *E*_*F*_ + *eV* (see Fig. 2b). So, for the applied bias *ε*_1_ < *eV* < *ε*_2_ occupation numbers ${N}_{1}^{T}({\varepsilon }_{1})$ and ${N}_{2}^{T}({\varepsilon }_{2})$ are close to 1/2 and 0 correspondingly and tunneling current through the single-electron states reads:

23 $${I}_{T}={I}_{1}\simeq \frac{8}{3}\cdot \frac{{\Gamma }_{L1}{\Gamma }_{R1}}{{\Gamma }_{L1}+{\Gamma }_{R1}} \sim \frac{4}{3}\cdot {\Gamma }_{L1}.$$

**Figure 4:**
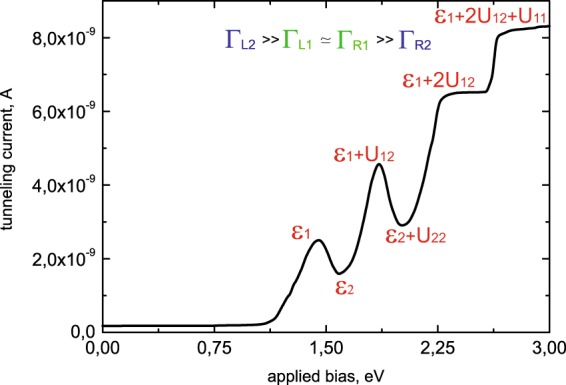
I-V characteristics for Γ_*L*2_ >> Γ_*L*1_ ≃ Γ_*R*1_ >> Γ_*R*2_. Calculation parameters are depicted by the red color.

Expression (23) reflects the growth of the tunneling current flowing through the impurity complex (see Fig. 4). Further increasing of applied bias (*ε*_2_ < *eV* < *ε*_1_ + *U*_12_) makes the second single -electron energy level available for tunneling (see Fig. 2c). Considering Eq. (20) and relations between the tunneling rates (22) one could find that occupation numbers are ${N}_{1}^{T}({\varepsilon }_{1})\simeq 1/2$, ${N}_{2}^{T}({\varepsilon }_{2})\simeq 1$. So, the tunneling current through the single-electron states now reads:

24 $${I}_{T}={I}_{2}\simeq 2\cdot \frac{{\Gamma }_{L2}{\Gamma }_{R2}}{{\Gamma }_{L2}+{\Gamma }_{R2}} \sim 2\cdot {\Gamma }_{R2} < \, < {I}_{1}.$$

Analyzing expression (24), the formation of negative conductivity could be revealed, as the growth of applied bias corresponds to the decrease of the tunneling current (see Fig. 4). It happens due to the influence of both Coulomb correlations and the asymmetry between the tunneling rates. Ratio (22) means that single-electron state *ε*_2_ fills much faster than the state *ε*_1_ and blocks tunneling through the energy level *ε*_1_ due to the Coulomb repulsion. Consequently, tunneling current value decreases.

For the higher values of applied bias (*ε*_1_ + *U*_12_ < *eV* < *ε*_2_ + *U*_2_) tunneling through the two-electron states becomes possible (see Fig. 2d). Now tunneling current is determined by the sum of Eqs. (20) and (21). Occupation numbers aspire to the values ${N}_{1}^{T}({\varepsilon }_{1})\simeq 1/2$, ${N}_{2}^{T}({\varepsilon }_{2})\simeq 1$, ${N}_{1}^{T}({\varepsilon }_{1}+{U}_{12})\simeq 1/2$ and ${N}_{2}^{T}({\varepsilon }_{2}+{U}_{2})\simeq 0$ and expression for the tunneling current through both single- and two-electron states (for our system parameters the lowest two-electron state has the energy *ε*_1_ + *U*_12_) takes the form (see Fig. 4):

25 $${I}_{T}\simeq \alpha \cdot {I}_{1}+{I}_{2}.$$

Parameter $\alpha =\frac{{\Gamma }_{L1}({\varepsilon }_{1}+{U}_{12})}{{\Gamma }_{L1}({\varepsilon }_{1})}\ge 1$ considers tunneling barrier transparency changing for the energy level *ε*_1_ + *U*_12_ as it is localized higher than the single-electron states. Further growth of applied bias (*ε*_2_ + *U*_2_ < *eV* < *ε*_1_ + 2*U*_12_) opens the possibility for the electrons with opposite spins to tunnel simultaneously through the two-electron state (see Fig. 2e). The tunneling rates asymmetry and the presence of Coulomb correlations cause blockade of the electrons, which tunnel through the single-electron state *ε*_1_. Occupation numbers aspire to the values ${N}_{1}^{T}({\varepsilon }_{1})\simeq 1/2$, ${N}_{2}^{T}({\varepsilon }_{2})\simeq 1$, ${N}_{1}^{T}({\varepsilon }_{1}+{U}_{12})\simeq 1/2$ and ${N}_{2}^{T}({\varepsilon }_{2}+{U}_{2})\simeq 1$ and tunneling current decreases. It is now determined only by the electrons tunneling through the two-electron state with the energy *ε*_2_ + *U*_22_:

26 $${I}_{T}\simeq 2\cdot {I}_{2} < \, < {I}_{1}+{I}_{2}.$$

Expression (26) corresponds to the formation of the second area with the negative conductivity, when the growth of applied bias results in the decrease of the tunneling current. The second minimum appears in the I-V curve (see Fig. 4). Further increasing of the applied bias (*ε*_1_ + 2*U*_12_ < *eV* < *ε*_1_ + *U*_1_ + 2*U*_12_) opens the possibility for electrons to tunnel through the three-electron states (see Fig. 2f). Occupation numbers ${N}_{1}^{T}({\varepsilon }_{1}+2{U}_{12})$ turn to 1/2, and expression for the current flowing through the system reads:

27 $${I}_{T}\simeq 2\cdot {I}_{2}+\alpha ^{\prime} {I}_{1},$$

where parameter $\alpha \text{'}=\frac{{\Gamma }_{L1}({\varepsilon }_{1}+2{U}_{12}}{{\Gamma }_{L1}({\varepsilon }_{1})}\ge 1$ takes into account tunneling barrier transparency changing for the three-electron states in comparison with the two-electron states. Further growth of applied bias leads to the formation of an additional step in the current voltage characteristic for *eV* > *ε*_1_ + *U*_1_ + 2*U*_12_, when tunneling through the four- electron states becomes possible (see Fig. 4).

We now consider the situation when strong asymmetry between the tunneling rates of the single electron energy level *ε*_1_ takes place:

28 $${\Gamma }_{L1} > \, > {\Gamma }_{L2}\simeq {\Gamma }_{R2} > \, > {\Gamma }_{R1}.$$

The behavior of I-V characteristics is quite similar to the previously discussed situation. The difference exists in the tunneling current values and the ranges of applied bias where negative conductivity appears.

**Figure 5:**
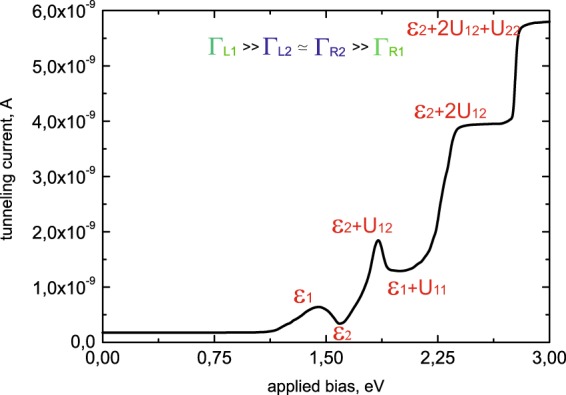
I-V characteristics for Γ_*L*1_ >> Γ_*L*2_ ≃ Γ_*R*2_ >> Γ_*R*1_. Calculation parameters are depicted by the red color.

For the small values of applied bias 0 < *eV* < *ε*_*i*_ (see Fig. 3a) tunneling current is negligibly small *I*_*T*_ → 0. The first maximum in the I-V characteristic (see Fig. 5) corresponds to the applied bias range *ε*_1_ < *eV* < *ε*_2_. The tunneling current amplitude is determined by the expression

29 $${I}_{T}={\tilde{I}}_{1}\simeq \frac{4}{2}\cdot \frac{{\Gamma }_{L1}{\Gamma }_{R1}}{{\Gamma }_{L1}+{\Gamma }_{R1}} \sim 2\cdot {\Gamma }_{R1}.$$

In this range of applied bias tunneling through the single-electron state with the lowest energy *ε*_1_ occurs (see Fig. 3b). The increasing of applied bias (*ε*_2_ < *eV* < *ε*_1_ + *U*_12_) opens the possibility for electrons to tunnel through both single-electron levels (see Fig. 3c). Consequently, current through the single-electron states reads:

30 $${I}_{T}\simeq \frac{1}{2}\cdot {\tilde{I}}_{1}.$$

Expression (30) reveals the formation of negative tunneling conductivity (see Fig. 5). It happens due to the presence of both Coulomb correlations and the asymmetry between the tunneling rates. As a results, the blockade of electron transport through the single-electron level *ε*_2_ takes place. For the higher values of applied bias (*ε*_2_ + *U*_12_ < *eV* < *ε*_1_ + *U*_1_) tunneling through the two-electron states becomes possible (see Fig. 3d). Tunneling current is then determined by the sum of Eqs. (20) and (21). It corresponds to the tunneling processes through the single-electron states and the two-electron states:

31 $${I}_{T}\simeq {\tilde{I}}_{1}+{\tilde{I}}_{2},$$

where ${\mathop{I}\limits^{ \sim }}_{2}\simeq {\Gamma }_{R2} > \, > {\mathop{I}\limits^{ \sim }}_{1}$. Figure 5 demonstrates growth of the tunneling current amplitude and formation of the second maximum in the I-V curve. Further increasing of the applied bias *(ε*_1_ + *U*_1_ < *eV* < *ε*_2_ + 2*U*_12_) leads to the simultaneous tunneling of electrons with opposite spins through the two-electron states (see Fig. 3e). Transport through the single-electron states is now blocked and the second area with the negative conductivity in the I-V characteristic appears (see Fig. 5):

32 $${I}_{T}\simeq 2\cdot {\tilde{I}}_{1}.$$

With the increasing of applied bias tunneling through the three- (see Fig. 3f) and four-electron states becomes possible and two additional steps are formed in the I-V curves.

We would like to mention, that in the absence of Coulomb interaction between localized electrons only steps would be seen in the I-V characteristics. The presence of strong Coulomb interaction significantly modifies I-V curves. In asymmetric contact areas with negative tunneling conductivity could arise (peaks are well pronounced at particular values of applied bias). The width of the peaks depends on the values of the tunneling rates. Tunneling rates values are determined by the the barriers widths and heights which are the functions of the particular geometry of the experiment and the symmetry of the localized states electronic orbitals. Moreover, tunneling barrier characteristics (width and height) are changed for the higher energy levels. As Coulomb interaction could be of order of several eV the barrier height for the high energy levels could be strongly changed.

Calculations demonstrated in Figs. 4 and 5 mostly correspond to the I-V curves available in the single-electron transistor geometry^4,39^. Typical current values obtained in the STM experiments are much smaller but all the features (peaks and steps) could be well resolved in both experimental schemes.

## Conclusions

To summarize, we studied the electron transport through the impurity cluster (modeled by the two-level system) localized between the tunneling contact leads taking into account strong Coulomb correlations and the asymmetry between the tunneling rates. By means of the generalized Keldysh diagram technique we derived general expressions for the tunneling current and obtained formulas, which define electron transport through the states with the different number of electrons. We demonstrated that the interplay between Coulomb correlations and the asymmetry between the tunneling rates result in the formation of multiple regions with negative tunneling conductivity in the I-V curves. We believe, that our results are very promising in the sense of single atoms transistors application in modern nanoelectronic circuits.

## Appendix

Expression for the tunneling current (15) includes mean electron occupation numbers ${n}_{i}^{\sigma }$, pair and triple correlation functions for the localized electrons, which have to be determined for a proper treatment of correlation effects. Equations for the total electron occupation numbers ${n}_{1}^{\sigma }$ and ${n}_{2}^{\sigma }$ can be found from the conditions:

33 $$\begin{array}{ccc}\frac{{\rm{\partial }}{n}_{1}^{\sigma }}{{\rm{\partial }}t} & = & {I}_{L1}^{\sigma }+{I}_{R1}^{\sigma }=0,\\ \frac{{\rm{\partial }}{n}_{2}^{\sigma }}{{\rm{\partial }}t} & = & {I}_{L2}^{\sigma }+{I}_{R2}^{\sigma }=0,\end{array}$$

where tunneling current ${I}_{L1}^{\sigma }$ reads:

34 $$\begin{array}{ccc}{I}_{L1}^{\sigma } & = & {\Gamma }_{L1}\times \{\langle {n}_{1}^{\sigma }\rangle -\langle (1-{n}_{1}^{-\sigma })(1-{n}_{2}^{-\sigma })(1-{n}_{2}^{\sigma })\cdot \rangle {N}_{L}^{\sigma }({\varepsilon }_{1})-\langle {n}_{1}^{-\sigma }(1-{n}_{2}^{-\sigma })(1-{n}_{2}^{\sigma })\rangle \cdot {N}_{L}^{\sigma }({\varepsilon }_{1}+{U}_{11})\\  &  & -\sum _{\sigma \text{'}}\,\langle {n}_{2}^{\sigma \text{'}}(1-{n}_{2}^{-\sigma \text{'}})(1-{n}_{1}^{-\sigma })\rangle \cdot {N}_{L}^{\sigma }({\varepsilon }_{1}+{U}_{12})-\sum _{\sigma \text{'}}\,\langle {n}_{1}^{-\sigma }{n}_{2}^{\sigma \text{'}}(1-{n}_{2}^{-\sigma })\rangle \cdot {N}_{L}^{\sigma }({\varepsilon }_{1}+{U}_{11}+{U}_{12})\\  &  & -\langle {n}_{2}^{\sigma }{n}_{2}^{-\sigma }(1-{n}_{1}^{-\sigma })\rangle \cdot {N}_{L}^{\sigma }({\varepsilon }_{1}+2{U}_{12})-\langle {n}_{1}^{-\sigma }{n}_{2}^{-\sigma }{n}_{2}^{\sigma }\rangle \cdot {N}_{L}^{\sigma }({\varepsilon }_{1}+{U}_{11}+2{U}_{12})\}.\end{array}$$

Tunneling current ${I}_{L2}^{\sigma }$ can be easily obtained from expression for the ${I}_{L1}^{\sigma }$ by the following indexes changing 1 ↔ 2 and ${I}_{Ri}^{\sigma }$ can be found by the indexes changing *L* ↔ *R*.

Pair correlation functions can be found as:

35 $$\langle \frac{\partial {n}_{i}^{\sigma }{n}_{j}^{{\sigma }^{\text{'}}}}{\partial t}\rangle =\langle \frac{\partial {n}_{i}^{\sigma }}{\partial t}\cdot {n}_{j}^{{\sigma }^{\text{'}}}\rangle +\langle \frac{\partial {n}_{j}^{{\sigma }^{\text{'}}}}{\partial t}\cdot {n}_{i}^{\sigma }\rangle =0$$

Full expressions for the pair correlation functions through the high order correlation functions have the form:

36 $$\begin{array}{c}({\Gamma }_{Li}+{\Gamma }_{Ri}+{\Gamma }_{Lj}+{\Gamma }_{Rj})\times \langle {n}_{i}^{\sigma }{n}_{j}^{{\sigma }^{\text{'}}}\rangle -\langle {n}_{j}^{{\sigma }^{\text{'}}}\rangle \times ({\Gamma }_{Li}\cdot {N}_{L}^{\sigma }({\varepsilon }_{i}+{U}_{ij})+{\Gamma }_{Ri}\cdot {N}_{R}^{\sigma }({\varepsilon }_{i}+{U}_{ij}))\\ -\,\langle {n}_{i}^{\sigma }\rangle \times [{\Gamma }_{Lj}\cdot {N}_{L}^{\sigma }({\varepsilon }_{j}+{U}_{ij})+{\Gamma }_{Rj}\cdot {N}_{R}^{\sigma }({\varepsilon }_{j}+{U}_{ij})]+\langle {n}_{i}^{\sigma }{n}_{i}^{-\sigma }\rangle \times [{\Gamma }_{Lj}\cdot {N}_{L}^{\sigma }({\varepsilon }_{j}+{U}_{ij})\\ +\,{\Gamma }_{Rj}\cdot {N}_{R}^{\sigma }({\varepsilon }_{j}+{U}_{ij})]-\langle {n}_{j}^{{\sigma }^{\text{'}}}{n}_{i}^{-\sigma }\rangle \times [-{\Gamma }_{Li}\cdot {N}_{L}^{\sigma }({\varepsilon }_{i}+{U}_{ij})-{\Gamma }_{Ri}\cdot {N}_{R}^{\sigma }({\varepsilon }_{i}+{U}_{ij})\\ +\,{\Gamma }_{Li}\cdot {N}_{L}^{\sigma }({\varepsilon }_{i}+{U}_{ii}+{U}_{ij})+{\Gamma }_{Ri}\cdot {N}_{R}^{\sigma }({\varepsilon }_{i}+{U}_{ii}+{U}_{ij})+{\Gamma }_{Lj}\cdot {N}_{L}^{\sigma }({\varepsilon }_{j}+2{U}_{ij})\\ +\,{\Gamma }_{Rj}\cdot {N}_{R}^{\sigma }({\varepsilon }_{j}+2{U}_{ij})]-\langle {n}_{j}^{{\sigma }^{\text{'}}}{n}_{j}^{-{\sigma }^{\text{'}}}\rangle \times [-{\Gamma }_{Li}\cdot {N}_{L}^{\sigma }({\varepsilon }_{i}+{U}_{ij})-{\Gamma }_{Ri}\cdot {N}_{R}^{\sigma }({\varepsilon }_{i}+{U}_{ij})\\ +\,{\Gamma }_{Li}\cdot {N}_{L}^{\sigma }({\varepsilon }_{i}+2{U}_{ij})+{\Gamma }_{Ri}\cdot {N}_{R}^{\sigma }({\varepsilon }_{i}+2{U}_{ij})]-\langle {n}_{j}^{-{\sigma }^{\text{'}}}{n}_{i}^{\sigma }\rangle \times [-{\Gamma }_{Lj}\cdot {N}_{L}^{\sigma }({\varepsilon }_{j}+{U}_{ij})\\ -\,{\Gamma }_{Rj}\cdot {N}_{R}^{\sigma }({\varepsilon }_{j}+{U}_{ij})+{\Gamma }_{Lj}\cdot {N}_{L}^{\sigma }({\varepsilon }_{j}+{U}_{jj}+2{U}_{ij})+{\Gamma }_{Rj}\cdot {N}_{R}^{\sigma }({\varepsilon }_{j}+{U}_{jj}+2{U}_{ij})]\\ -\,\langle {n}_{j}^{{\sigma }^{\text{'}}}{n}_{j}^{-{\sigma }^{\text{'}}}{n}_{i}^{-\sigma }\rangle \times [{\Gamma }_{Li}\cdot {N}_{L}^{\sigma }({\varepsilon }_{i}+{U}_{ij})+{\Gamma }_{Ri}\cdot {N}_{R}^{\sigma }({\varepsilon }_{i}+{U}_{ij})-{\Gamma }_{Li}\cdot {N}_{L}^{\sigma }({\varepsilon }_{i}+{U}_{ii}+{U}_{ij})\\ -\,{\Gamma }_{Ri}\cdot {N}_{R}^{\sigma }({\varepsilon }_{i}+{U}_{ii}+{U}_{ij})-{\Gamma }_{Li}\cdot {N}_{L}^{\sigma }({\varepsilon }_{i}+2{U}_{ij})-{\Gamma }_{Ri}\cdot {N}_{R}^{\sigma }({\varepsilon }_{i}+2{U}_{ij})\\ -\,{\Gamma }_{Li}\cdot {N}_{L}^{\sigma }({\varepsilon }_{i}+{U}_{ii}+2{U}_{ij})-{\Gamma }_{Ri}\cdot {N}_{R}^{\sigma }({\varepsilon }_{i}+{U}_{ii}+2{U}_{ij})-{\Gamma }_{Lj}\cdot {N}_{L}^{\sigma }({\varepsilon }_{j}+{U}_{jj}+{U}_{ij})\\ -\,{\Gamma }_{Rj}\cdot {N}_{R}^{\sigma }({\varepsilon }_{j}+{U}_{jj}+{U}_{ij})]-\langle {n}_{i}^{\sigma }{n}_{i}^{-\sigma }{n}_{j}^{-{\sigma }^{\text{'}}}\rangle \times [{\Gamma }_{Lj}\cdot {N}_{L}^{\sigma }({\varepsilon }_{j}+{U}_{ij})+{\Gamma }_{Rj}\cdot {N}_{R}^{\sigma }({\varepsilon }_{j}+{U}_{ij})\\ -\,{\Gamma }_{Lj}\cdot {N}_{L}^{\sigma }({\varepsilon }_{j}+{U}_{jj}+{U}_{ij})-{\Gamma }_{Rj}\cdot {N}_{R}^{\sigma }({\varepsilon }_{j}+{U}_{jj}+{U}_{ij})+{\Gamma }_{Lj}\cdot {N}_{L}^{\sigma }({\varepsilon }_{j}+{U}_{jj}+2{U}_{ij})\\ +\,{\Gamma }_{Rj}\cdot {N}_{R}^{\sigma }({\varepsilon }_{j}+{U}_{jj}+2{U}_{ij})]=0.\end{array}$$

Third order correlation functions can be found in the similar way:

37 $$\langle \frac{\partial {n}_{j}^{\sigma }{n}_{j}^{-\sigma }{n}_{i}^{-\sigma \text{'}}}{\partial t}\rangle =\langle \frac{\partial {n}_{j}^{\sigma }{n}_{j}^{-\sigma }}{\partial t}\cdot {n}_{i}^{-\sigma \text{'}}\rangle +\langle \frac{\partial {n}_{i}^{-\sigma \text{'}}}{\partial t}\cdot {n}_{j}^{\sigma }{n}_{j}^{-\sigma }\rangle =0$$

So, expressions for the third order correlation functions have the form:

38 $$\begin{array}{c}\langle {n}_{j\sigma }{n}_{j-\sigma }{n}_{i-{\sigma }^{\text{'}}}\rangle -\frac{[\langle {n}_{i}^{-\sigma }{n}_{j}^{\sigma }\rangle +\langle {n}_{i}^{-\sigma }{n}_{j}^{-\sigma }\rangle ]\cdot [{\Gamma }_{Lj}\cdot {N}_{L}^{\sigma }({\varepsilon }_{j}+{U}_{jj}+2{U}_{ij})+{\Gamma }_{Rj}\cdot {N}_{R}^{\sigma }({\varepsilon }_{j}+{U}_{jj}+{U}_{2ij})]}{{\Gamma }_{Li}\cdot [3+{N}_{L}^{\sigma }({\varepsilon }_{i}+2{U}_{ij})-{N}_{L}^{\sigma }({\varepsilon }_{i}+{U}_{ii}+2{U}_{ij})]+{\Gamma }_{Ri}\cdot [3+{N}_{R}^{\sigma }({\varepsilon }_{i}+2{U}_{ij})-{N}_{R}^{\sigma }({\varepsilon }_{i}+{U}_{ii}+2{U}_{ij})]}\\ +\,\frac{\langle {n}_{j}^{\sigma }{n}_{j}^{-\sigma }\rangle \cdot [{\Gamma }_{Lj}\cdot {N}_{L}^{\sigma }({\varepsilon }_{i}+2{U}_{ij})+{\Gamma }_{Ri}\cdot {N}_{R}^{\sigma }({\varepsilon }_{i}{U}_{2ij})]}{{\Gamma }_{Li}\cdot [3+{N}_{L}^{\sigma }({\varepsilon }_{i}+2{U}_{ij})-{N}_{L}^{\sigma }({\varepsilon }_{i}+{U}_{ii}+2{U}_{ij})]+{\Gamma }_{Ri}\cdot [3+{N}_{R}^{\sigma }({\varepsilon }_{i}+2{U}_{ij})-{N}_{R}^{\sigma }({\varepsilon }_{i}+{U}_{ii}+2{U}_{ij})]}=0.\end{array}$$
